# Supplementary material for: The effect of entrepreneurship education on the entrepreneurial intention of different college students: Gender, household registration, school type, and poverty status
Source: PLoS One. 2023 Jul 19;18(7):e0288825. doi: 10.1371/journal.pone.0288825 (PMC10355450; doi:10.1371/journal.pone.0288825)
Supplement: S1 Data — (ZIP) [file pone.0288825.s001.zip › Data_procedure_tables_revised_R2/entrepreneurship_table_1_11_R2.docx]

The effect of entrepreneurship education on the entrepreneurial intention of different college students: Gender, household registration, school type, and poverty status

====================================================================

| **Table 1. Definition of variables** | |
| --- | --- |
| **Variable** | **Definition** |
| **Entrepreneurship** **intention** | Whether you are likely to start a business (0=no, 1=yes) |
| **education** | Whether the school's EE has increased your knowledge and ability of entrepreneurship (1=strongly disagree, 2=more disagree, 3=medium, 4=more agree, 5=strongly agree) |
| **age** | Age range from 19 to 27 |
| **gender** | Gender (0=female, 1=male) |
| **ethnicity** | Ethnicity (0=ethnic minorities, 1=Han nationality) |
| **hukou** | Household registration (0=urban, 1=rural) |
| **region** | Area of residence (1=western region, 2=central Region, 3=eastern region) |
| **party** | Whether you intend to join the party  (0=no, 1=yes) |
| **family_economic** | The level of the economic status of the family in the location (1=well below average, 2=below average, 3=average, 4=above average, 5=well above average) |
| **siblings** | Number of siblings (1=zero, 2=one, 3=two, 4=three,  5=four or more) |
| **father_edu_year** | Years of education of the father |
| **father_employ** | Whether the father has his own business (0=no, 1=yes) |
| **mother_employ** | Whether the mother has her own business  (0=no, 1=yes) |
| **father_enterprise** | Do your parents want you to start your own business?  (1=very much not hopeful, 2=some do not hopeful, 3=medium, 4=Some hopeful, 5=very hopeful) |
| **major_type** | Types of personnel training  (1=general undergraduate, 2=vocational bachelor's degree,  3=junior college starting point to undergraduate degree) |
| **grade** | Grade level (1=freshman, 2=sophomore, 3=junior, 4=senior) |
| **class** | There are 16 classes in total. |
| **learn** | Academic achievement grade (1=very bad, 2=a little bad, 3=medium, 4=good, 5=excellent) |
| **student_leader** | Served as a student cadre (0=no, 1=yes) |
| **student_club** | Joined a student club (0=no, 1=yes) |
| **volunteer** | Participated in a student volunteer organization  (0=no, 1=yes) |
| **parttime_job** | Part-time work experience (0=no, 1=yes) |
| **internship** | Internship experience (0=no, 1=yes) |

| **Table 2. Statistical description of variables** | | | | | | |
| --- | --- | --- | --- | --- | --- | --- |
| **Variable** | **Obs** | **Mean** | **SD** | **Min** | **Median** | **Max** |
| **Entrepreneurship intention** | 518 | 0.4633 | 0.4991 | 0 | 0 | 1 |
| **education** | 518 | 3.2317 | 0.7894 | 1 | 3 | 5 |
| **age** | 518 | 21.7220 | 1.5374 | 19 | 22 | 27 |
| **gender** | 518 | 0.7336 | 0.4425 | 0 | 1 | 1 |
| **ethnicity** | 518 | 0.7046 | 0.4566 | 0 | 1 | 1 |
| **hukou** | 518 | 0.8069 | 0.3951 | 0 | 1 | 1 |
| **region** | 518 | 1.3436 | 0.6506 | 1 | 1 | 3 |
| **party** | 518 | 0.3977 | 0.4899 | 0 | 0 | 1 |
| **family_economic** | 518 | 2.5347 | 0.6854 | 1 | 3 | 5 |
| **siblings** | 518 | 2.4595 | 1.0850 | 1 | 2 | 5 |
| **father_edu_year** | 518 | 9.2934 | 2.6342 | 0 | 9 | 19 |
| **father_employ** | 518 | 0.1795 | 0.3842 | 0 | 0 | 1 |
| **mother_employ** | 518 | 0.1429 | 0.3503 | 0 | 0 | 1 |
| **father_enterprise** | 518 | 2.9788 | 0.8142 | 1 | 3 | 5 |
| **major** | 518 | 1.7181 | 1.1408 | 1 | 1 | 4 |
| **major_type** | 518 | 1.7780 | 0.7379 | 1 | 2 | 3 |
| **grade** | 518 | 2.7432 | 0.9400 | 1 | 3 | 4 |
| **class** | 518 | 9.1274 | 4.7939 | 1 | 10 | 16 |
| **learn** | 518 | 3.1795 | 0.7969 | 1 | 3 | 5 |
| **student_leader** | 518 | 0.5347 | 0.4993 | 0 | 1 | 1 |
| **student_club** | 518 | 0.7819 | 0.4134 | 0 | 1 | 1 |
| **volunteer** | 518 | 0.7529 | 0.4317 | 0 | 1 | 1 |
| **parttime_job** | 518 | 0.6757 | 0.4686 | 0 | 1 | 1 |
| **internship** | 518 | 0.4768 | 0.4999 | 0 | 0 | 1 |

| **Table 3. The impact of EE on the EI of college students: Benchmark regression** | | | | |
| --- | --- | --- | --- | --- |
| **Variable** | **Dependent variable: Entrepreneurship intention** | | | |
|  | **(1)** | **(2)** | **(3)** | **(4)** |
| **education** | 2.1217*** | 2.0246*** | 1.9951*** | 1.8634*** |
|  | (0.2739) | (0.2631) | (0.2641) | (0.2495) |
| **age** |  | 1.0037 | 0.9943 | 1.0006 |
|  |  | (0.0618) | (0.0637) | (0.1089) |
| **gender** |  | 0.9165 | 0.7862 | 0.7455 |
|  |  | (0.1984) | (0.1765) | (0.1797) |
| **ethnicity** |  | 0.8775 | 0.8569 | 0.8488 |
|  |  | (0.1824) | (0.1831) | (0.1882) |
| **hukou** |  | 1.2824 | 1.1338 | 1.0762 |
|  |  | (0.3094) | (0.3051) | (0.2972) |
| **region** |  | 0.6988** | 0.7254* | 0.8445 |
|  |  | (0.1104) | (0.1208) | (0.1580) |
| **party** |  | 0.8181 | 0.8220 | 0.7582 |
|  |  | (0.1601) | (0.1661) | (0.1634) |
| **family_economic** |  |  | 0.9744 | 0.9918 |
|  |  |  | (0.1460) | (0.1516) |
| **siblings** |  |  | 0.8892 | 0.8885 |
|  |  |  | (0.0844) | (0.0863) |
| **father_edu_year** |  |  | 0.9540 | 0.9574 |
|  |  |  | (0.0386) | (0.0397) |
| **father_employ** |  |  | 0.9371 | 0.9080 |
|  |  |  | (0.3007) | (0.3016) |
| **mother_employ** |  |  | 0.8987 | 0.9331 |
|  |  |  | (0.3065) | (0.3330) |
| **father_enterprise** |  |  | 1.7998*** | 1.7233*** |
|  |  |  | (0.2317) | (0.2259) |
| **major** |  |  |  | 1.1627 |
|  |  |  |  | (0.3537) |
| **major_type** |  |  |  | 0.7045 |
|  |  |  |  | (0.1803) |
| **grade** |  |  |  | 0.6029 |
|  |  |  |  | (0.2067) |
| **class** |  |  |  | 0.8343* |
|  |  |  |  | (0.0854) |
| **learn** |  |  |  | 1.0345 |
|  |  |  |  | (0.1347) |
| **student_leader** |  |  |  | 1.2677 |
|  |  |  |  | (0.2737) |
| **student_club** |  |  |  | 1.6387* |
|  |  |  |  | (0.4263) |
| **volunteer** |  |  |  | 0.8278 |
|  |  |  |  | (0.2139) |
| **parttime_job** |  |  |  | 1.1077 |
|  |  |  |  | (0.2508) |
| **internship** |  |  |  | 0.9972 |
|  |  |  |  | (0.2407) |
| **Constant** | 0.0746*** | 0.1334 | 0.0806 | 1.4688 |
|  | (0.0323) | (0.1971) | (0.1333) | (4.1869) |
| **Observations** | 518 | 518 | 518 | 518 |
| **Pseudo R-squared** | 0.0544 | 0.0691 | 0.1030 | 0.1260 |
|  | | | | |
| Note: Regression values are odds ratios. The values in parentheses are standard errors. *** p<0.01, ** p<0.05, * p<0.1 | | | | |

| **Table 4. Replacing the independent variable：Robustness test** | | | | |
| --- | --- | --- | --- | --- |
| **Variable** | **Dependent variable: Entrepreneurship intention** | | | |
|  | **(1)** | **(2)** | **(3)** | **(4)** |
| **edu_necessary** | 1.7031*** | 1.6743*** | 1.6200*** | 1.4791*** |
|  | (0.1831) | (0.1829) | (0.1796) | (0.1697) |
| **Individual** |  | Yes | Yes | Yes |
| **Family** |  |  | Yes | Yes |
| **School** |  |  |  | Yes |
| **Constant** | 0.1219*** | 0.2788 | 0.1756 | 2.0123 |
|  | (0.0497) | (0.4022) | (0.2832) | (5.6707) |
| **Observations** | 518 | 518 | 518 | 518 |
| **Pseudo R-squared** | 0.0375 | 0.0564 | 0.0892 | 0.1110 |
|  | | | | |
| Note: Regression values are odds ratios. The values in parentheses are standard errors. *** p<0.01, ** p<0.05, * p<0.1 | | | | |

| **Table 5. Deleting the sample of undergraduate students from junior college： Robustness test** | | | | |
| --- | --- | --- | --- | --- |
| **Variable** | **Dependent variable: Entrepreneurship intention** | | | |
|  | **(1)** | **(2)** | **(3)** | **(4)** |
| **education** | 2.4060*** | 2.2553*** | 2.2023*** | 2.0957*** |
|  | (0.3579) | (0.3417) | (0.3405) | (0.3371) |
| **Individual** |  | Yes | Yes | Yes |
| **Family** |  |  | Yes | Yes |
| **School** |  |  |  | Yes |
| **Constant** | 0.0449*** | 5.507 | 2.8491 | 224.1388 |
|  | (0.0226) | (11.4075) | (6.4837) | (800.3093) |
| **Observations** | 422 | 422 | 422 | 422 |
| **Pseudo R-squared** | 0.0714 | 0.0982 | 0.1250 | 0.1490 |
|  | | | | |
| Note: Regression values are odds ratios. The values in parentheses are standard errors. *** p<0.01, ** p<0.05, * p<0.1 | | | | |

| **Table 6. Deleting the samples with the best and worst academic performance： Robustness test** | | | | |
| --- | --- | --- | --- | --- |
| **Variable** | **Dependent variable: Entrepreneurship intention** | | | |
|  | **(1)** | **(2)** | **(3)** | **(4)** |
| **education** | 2.3923*** | 2.2512*** | 2.1938*** | 2.0138*** |
|  | (0.3414) | (0.3260) | (0.3269) | (0.3032) |
| **Individual** |  | Yes | Yes | Yes |
| **Family** |  |  | Yes | Yes |
| **School** |  |  |  | Yes |
| **Constant** | 0.0483*** | 0.1060 | 0.0430* | 0.2778 |
|  | (0.0232) | (0.1675) | (0.0763) | (0.8566) |
| **Observations** | 483 | 483 | 483 | 483 |
| **Pseudo R-squared** | 0.0656 | 0.0845 | 0.1230 | 0.1430 |
|  |  |  |  |  |
| Note: Regression values are odds ratios. The values in parentheses are standard errors. *** p<0.01, ** p<0.05, * p<0.1 | | | | |

| **Table 7. Changing the empirical model：Robustness test** | | | | |
| --- | --- | --- | --- | --- |
| **Variable** | **Dependent variable: Entrepreneurship intention** | | | |
|  | **LPM** | | **Probit** | |
|  | **(1)** | **(2)** | **(3)** | **(4)** |
| **education** | 0.1688*** | 0.1289*** | 0.1745*** | 0.1478*** |
|  | (0.0268) | (0.0273) | (0.0290) | (0.0310) |
| **Individual** |  | Yes |  | Yes |
| **Family** |  | Yes |  | Yes |
| **School** |  | Yes |  | Yes |
| **Constant** | -0.0823 | 0.5471 |  |  |
|  | (0.0892) | (0.6081) |  |  |
| **Observations** | 518 | 518 | 518 | 518 |
| **R-squared** | 0.0713 | 0.1590 |  |  |
| **Pseudo R-squared** |  |  | 0.0525 | 0.1270 |
|  |  |  |  |  |
| Note: The regression values of the LPM are the coefficient values. The regression value of the Probit is the marginal effect. The values in parentheses are standard errors. *** p<0.01, ** p<0.05, * p<0.1 | | | | |

| **Table 8. Gender differences** | | | | |
| --- | --- | --- | --- | --- |
| **Variable** | **Dependent variable: Entrepreneurship intention** | | | |
|  | **(1)** | **(2)** | **(3)** | **(4)** |
| **Panel A: Male** |  | | | |
| **education** | 2.0402*** | 1.9981*** | 2.0039*** | 1.8355*** |
|  | (0.3051) | (0.3004) | (0.3124) | (0.2949) |
| **Individual** |  | Yes | Yes | Yes |
| **Family** |  |  | Yes | Yes |
| **School** |  |  |  | Yes |
| **Constant** | 0.0826*** | 0.0946 | 0.0556 | 0.8506 |
|  | (0.0412) | (0.1593) | (0.1055) | (2.7850) |
| **Observations** | 380 | 380 | 380 | 380 |
| **Pseudo R-squared** | 0.0491 | 0.0673 | 0.1110 | 0.1400 |
| **Panel B: Female** |  | | | |
| **education** | 2.3413*** | 2.1674*** | 2.0822*** | 2.3192*** |
|  | (0.6029) | (0.5720) | (0.5561) | (0.6578) |
| **Individual** |  | Yes | Yes | Yes |
| **Family** |  |  | Yes | Yes |
| **School** |  |  |  | Yes |
| **Constant** | 0.0576*** | 0.6924 | 0.2061 | 1.4037 |
|  | (0.0506) | (2.1159) | (0.7360) | (9.0559) |
| **Observations** | 138 | 138 | 138 | 138 |
| **Pseudo R-squared** | 0.0680 | 0.0882 | 0.1060 | 0.1480 |
| **Group difference** | 0.1377 | 0.0814 | 0.0383 | 0.2339 |
|  |  |  |  |  |
| Note: Regression values are odds ratios. The values in parentheses are standard errors. Differences in coefficients between groups were tested using Fisher's bootstrap based (1000 times) approach. *** p<0.01, ** p<0.05, * p<0.1 | | | | |

| **Table 9. Differences between urban and rural college students** | | | | |
| --- | --- | --- | --- | --- |
| **Variable** | **Dependent variable: Entrepreneurship intention** | | | |
|  | **(1)** | **(2)** | **(3)** | **(4)** |
| **Panel A: Rural** |  | | | |
| **education** | 1.7994*** | 1.7371*** | 1.6726*** | 1.5539*** |
|  | (0.2446) | (0.2382) | (0.2360) | (0.2255) |
| **Individual** |  | Yes | Yes | Yes |
| **Family** |  |  | Yes | Yes |
| **School** |  |  |  | Yes |
| **Constant** | 0.1364*** | 0.2704 | 0.0984 | 1.8078 |
|  | (0.0623) | (0.4283) | (0.1735) | (5.6205) |
| **Observations** | 418 | 418 | 418 | 418 |
| **Pseudo R-squared** | 0.0354 | 0.0454 | 0.0831 | 0.1160 |
| **Panel B: Urban** |  | | | |
| **education** | 5.8870*** | 7.2027*** | 10.3025*** | 12.0659*** |
|  | (2.4457) | (3.3875) | (5.6590) | (8.1387) |
| **Individual** |  | Yes | Yes | Yes |
| **Family** |  |  | Yes | Yes |
| **School** |  |  |  | Yes |
| **Constant** | 0.0020*** | 0.0036 | 0.0344 | 0.0025 |
|  | (0.0028) | (0.0162) | (0.1813) | (0.0245) |
| **Observations** | 100 | 100 | 100 | 100 |
| **Pseudo R-squared** | 0.1990 | 0.2440 | 0.3250 | 0.3960 |
| **Group difference** | 1.1853** | 1.4222*** | 1.8180*** | 2.0496 *** |
|  |  |  |  |  |
| Note: Regression values are odds ratios. The values in parentheses are standard errors. Differences in coefficients between groups were tested using Fisher's bootstrap based (1000 times) approach. *** p<0.01, ** p<0.05, * p<0.1 | | | | |

| **Table 10. Differences between public and private universities** | | | | |
| --- | --- | --- | --- | --- |
| **Variable** | **Dependent variable: Entrepreneurship intention** | | | |
|  | **(1)** | **(2)** | **(3)** | **(4)** |
| **Panel A: Public** |  | | | |
| **education** | 1.5432*** | 1.5477*** | 1.5709*** | 1.4744** |
|  | (0.2171) | (0.2196) | (0.2307) | (0.2223) |
| **Individual** |  | Yes | Yes | Yes |
| **Family** |  |  | Yes | Yes |
| **School** |  |  |  | Yes |
| **Constant** | 0.2609*** | 0.2020** | 0.1991 | 2.7067 |
|  | (0.1248) | (0.1462) | (0.2045) | (6.5741) |
| **Observations** | 350 | 350 | 350 | 350 |
| **Pseudo R-squared** | 0.0207 | 0.0238 | 0.0705 | 0.0932 |
| **Panel B: Private** |  | | | |
| **education** | 5.2519*** | 5.2584*** | 5.0957*** | 6.7819*** |
|  | (1.7284) | (1.7746) | (1.7774) | (2.8120) |
| **Individual** |  | Yes | Yes | Yes |
| **Family** |  |  | Yes | Yes |
| **School** |  |  |  | Yes |
| **Constant** | 0.0027*** | 0.0074*** | 0.0011*** | 0.1042 |
|  | (0.0029) | (0.0096) | (0.0022) | (0.8751) |
| **Observations** | 168 | 168 | 168 | 168 |
| **Pseudo R-squared** | 0.1730 | 0.1980 | 0.2450 | 0.3450 |
| **Group difference** | 1.2247 *** | 1.2231*** | 1.1767*** | 1.5260 *** |
|  |  |  |  |  |
| Note: Regression values are odds ratios. The values in parentheses are standard errors. Differences in coefficients between groups were tested using Fisher's bootstrap based (1000 times) approach. *** p<0.01, ** p<0.05, * p<0.1 | | | | |

| **Table 11. Differences between poverty and nonpoverty college students** | | | | |
| --- | --- | --- | --- | --- |
| **Variable** | **Dependent variable: Entrepreneurship intention** | | | |
|  | **(1)** | **(2)** | **(3)** | **(4)** |
| **Panel A: Poverty** |  | | | |
| **education** | 1.4580* | 1.4988* | 1.5282** | 1.5114* |
|  | (0.2897) | (0.3148) | (0.3245) | (0.3249) |
| **Individual** |  | Yes | Yes | Yes |
| **Family** |  |  | Yes | Yes |
| **School** |  |  |  | Yes |
| **Constant** | 0.2938* | 0.0987 | 0.1831 | 9.9460 |
|  | (0.1936) | (0.2631) | (0.4969) | (42.7518) |
| **Observations** | 169 | 169 | 169 | 169 |
| **Pseudo R-squared** | 0.0160 | 0.0397 | 0.0471 | 0.0706 |
| **Panel B: Nonpoverty** |  | | | |
| **education** | 2.6986*** | 2.6531*** | 2.6200*** | 2.4226*** |
|  | (0.4628) | (0.4674) | (0.4643) | (0.4350) |
| **Individual** |  | Yes | Yes | Yes |
| **Family** |  |  | Yes | Yes |
| **School** |  |  |  | Yes |
| **Constant** | 0.0312*** | 0.1322 | 0.1160 | 4.1364 |
|  | (0.0181) | (0.2407) | (0.2206) | (14.7584) |
| **Observations** | 349 | 349 | 349 | 349 |
| **Pseudo R-squared** | 0.0852 | 0.1090 | 0.1090 | 0.1280 |
| **Group difference** | 0.6156** | 0.5711** | 0.5391** | 0.4718* |
|  |  |  |  |  |
| Note: Regression values are odds ratios. The values in parentheses are standard errors. Differences in coefficients between groups were tested using Fisher's bootstrap based (1000 times) approach. *** p<0.01, ** p<0.05, * p<0.1 | | | | |
