# Supplementary material for: The effect of entrepreneurship education on the entrepreneurial intention of different college students: Gender, household registration, school type, and poverty status
Source: PLoS One. 2023 Jul 19;18(7):e0288825. doi: 10.1371/journal.pone.0288825 (PMC10355450; doi:10.1371/journal.pone.0288825)
Supplement: Attachment — Submitted filename: Review.docx [file pone.0288825.s002.docx]

Review

Thank you for the invitation to read and review this article, here are my views for reference.

The paper has done a lot of research and analysis work, the analysis procedure is detailed, and the conclusions also have a good practical reference role.

Limitations:

1. This paper is not innovative enough and has made little academic contribution. There are many articles on the impact of entrepreneurship education on entrepreneurial intentions, and articles on factors such as gender and economic conditions have a large number of literature.
2. The data sources of the paper sample are relatively concentrated and lack representativeness.
3. Line 744 says "fill the research gap" and line 817 says "extends.... for the theory", exaggerating the significance and theoretical contribution of this study. In addition, page 17 Table 1. Definition of variables, "feconomic", "fatherduyr", " Is the word "fenterprise" misspelled? Lines 100-101, uniform format for numbers after section: Section II, Section 3.
4. The language of the paper needs polishing.
