# Supplementary material for: The effect of entrepreneurship education on the entrepreneurial intention of different college students: Gender, household registration, school type, and poverty status
Source: PLoS One. 2023 Jul 19;18(7):e0288825. doi: 10.1371/journal.pone.0288825 (PMC10355450; doi:10.1371/journal.pone.0288825)
Supplement: Attachment — Submitted filename: Response to Reviewers.docx [file pone.0288825.s004.docx]

==================================================

**Response to the comments of the editor and reviewers**

==================================================

Dear editor and reviewers,

Thank you for your letter and the reviewers' comments on our manuscript entitled "**The effect of entrepreneurship education on the entrepreneurial intention of different college students: Gender, household registration, school type, and poverty status**" (ID: **PONE-D-23-03086R1**). All of these comments were very helpful for revising and improving our paper. We have carefully studied these comments and made corrections, which we hope you will approve.

The changes in the revised manuscript are marked in **red**.

The point-to-point responses to comments from the editor and reviewers are provided below.

=================================================

**Response to Journal Requirements**

**Comment:** *Please review your reference list to ensure that it is complete and correct. If you have cited papers that have been retracted, please include the rationale for doing so in the manuscript text, or remove these references and replace them with relevant current references. Any changes to the reference list should be mentioned in the rebuttal letter that accompanies your revised manuscript. If you need to cite a retracted article, indicate the article's retracted status in the References list and also include a citation and full reference for the retraction notice.*

**Response:** We have carefully checked to ensure that the list of references in this paper is complete and correct. First, this paper does not cite the retracted papers. Second, compared with the first submission, seven kinds of literature were added to the first revised draft (ID: PONE-D-23-03086R1), while no literature was added to this revised draft. Third, the list of seven additional papers is as follows (note: the numbers in [] are the serial numbers of the papers in the article) :

[88] Dana L-P, Tajpour M, Salamzadeh A, Hosseini E, Zolfaghari M. The impact of entrepreneurial education on technology-based enterprises development: The mediating role of motivation. Administrative Sciences. 2021;11(4):105.

[93] Salamzadeh A, Tajpour M, Hosseini E. Measuring the impact of simulation-based teaching on entrepreneurial skills of the MBA/DBA students. Technology and Entrepreneurship Education: Adopting Creative Digital Approaches to Learning and Teaching: Springer; 2022. p. 77-104.

[94] Salamzadeh Y, Sangosanya TA, Salamzadeh A, Braga V. Entrepreneurial universities and social capital: The moderating role of entrepreneurial intention in the Malaysian context. International Journal of Management Education. 2022;20(1). doi: 10.1016/j.ijme.2022.100609. PubMed PMID: WOS:000760340200006.

[100] Salamzadeh A, Farjadian AA, Amirabadi M, Modarresi M. Entrepreneurial characteristics: Insights from undergraduate students in Iran. International Journal of Entrepreneurship Small Business. 2014;21(2):165-82.

[103] Rahman MM, Salamzadeh A, Tabash M. Antecedents of entrepreneurial intentions of female undergraduate students in Bangladesh: A covariance-based structural equation modeling approach. Journal of Women's Entrepreneurship and Education. 2022;(1-2):137-53. doi: 10.28934/jwee22.12.pp137-153.

[107] Ramadani V, Rahman MM, Salamzadeh A, Rahaman MS, Abazi-Alili H. Entrepreneurship education and graduates' entrepreneurial intentions: Does gender matter? A multi-group analysis using AMOS. Technological Forecasting Social Change. 2022;180:121693.

[110] Assari S. Parental Education Attainment and Educational Upward Mobility; Role of Race and Gender[J]. Behavioral Sciences, 2018, 8(11).

==============================================

**Response to Reviewer #2**

**Comment 2:** *You have put a reasonable effort into minimizing the mistakes and improving the paper substantially. The new version of the paper has added two other parts to enhance the theoretical parts while trying to expand the previous version's theoretical underpinnings and practical implications. In the current version, I can see justifications for how the selected theoretical stance can be an appropriate lens for studying the issue in question. Given the level of effort that has been put into the new version of the paper and due to the paper's relevance to the topic of the issue, I am satisfied with the current version of the article.*

**Response 2:** Thank you for your insightful comments and warm encouragement.

===============================================

**Response to Reviewer #4**

**Comment 4.1:** *The paper is clearly structured. From the overall presentation I would say that interesting research work has been done. The topic is also important for the readers of the journal. In the revised version, the manuscript has been extended and improved.*

**Response 4.1:** Thank you for your insightful comments and warm encouragement.

**Comment 4.2:** *The dependent variable is the students' EI (entrepreneurial intention). Therefore, entrepreneurial intention has to be included as dependent variable in all Tables (not "Entrepreneurship").*

**Response 4.2:** Thank you for your careful advice. We have changed the dependent variable in all tables to entrepreneurial intention. Changes in the manuscript are marked in **red**.

===================================================
